# Fatty Acid Compositions of Six Wild Edible Mushroom Species

**DOI:** 10.1155/2013/163964

**Published:** 2013-06-06

**Authors:** Pelin Günç Ergönül, Ilgaz Akata, Fatih Kalyoncu, Bülent Ergönül

**Affiliations:** ^1^Department of Food Engineering, Faculty of Engineering, Celal Bayar University, 45140 Manisa, Turkey; ^2^Department of Biology, Faculty of Science, Ankara University, Tandogan, 06100 Ankara, Turkey; ^3^Department of Biology, Faculty of Science and Letters, Celal Bayar University, 45140 Manisa, Turkey

## Abstract

The fatty acids of six wild edible mushroom species (*Boletus reticulatus*, *Flammulina velutipes* var. *velutipes*, *Lactarius salmonicolor*, *Pleurotus ostreatus*, *Polyporus squamosus,* and *Russula anthracina*) collected from different regions from Anatolia were determined. The fatty acids were identified and quantified by gas chromatography and studied using fruit bodies. Fatty acid composition varied among species. The dominant fatty acid in fruit bodies of all mushrooms was *cis*-linoleic acid (18 : 2). Percentage of *cis*-linoleic acid in species varied from 22.39% to 65.29%. The other major fatty acids were, respectively, *cis*-oleic, palmitic, and stearic acids. Fatty acids analysis of the mushrooms showed that the unsaturated fatty acids were at higher concentrations than saturated fatty acids.

## 1. Introduction

More than 2000 species of mushrooms exist in nature; however less than 25 species are widely accepted as food and only a few have attained the level of an item of commerce. Wild mushrooms are becoming more and more important in our diet for their nutritional, organoleptic, and pharmacological characteristics [1]. Based on their high mineral content, low fat, and low calories, the nutritional values of mushrooms have already been reevaluated [2]. Additionally, mushrooms have been known to have an effect on preventing several diseases such as cancer, hypercholesterolemia, and hypertension [3]. 

Dietary fat, a major constituent of the normal diet and thus a tight feedback regulator, is necessary to ensure balanced lipid homeostasis. Generally, lipid content of mushroom species is low. It is reported that, in fresh mushrooms belonging to different species, the lipid proportion per 100 g is 1.75–15.5% in dried mushroom since fresh ones contain high amounts of water [4]. Although the edible wild mushrooms command higher prices than cultivated mushrooms, people prefer to consume them due to their flavour and texture [5]. Therefore, it is necessary to investigate the levels of chemical and biochemical compounds in wild edible mushrooms, because many wild edible mushroom species are known to store high levels of several unsaturated fatty acids [6]. 

Many researchers have studied the fatty acid composition of several mushrooms and elucidated their nutritional roles in the human diet [7]. Barros et al. [1] reported that the major fatty acids of *Agaricus arvensis*, *Lactarius deliciosus*, *Leucopaxillus giganteus*, *Sarcodon imbricatus,* and *Tricholoma portentosum* were linoleic acid and oleic acid. *Cordyceps sinensis* is known to contain 16 kinds of amino acids, and 27.4% of its total fatty acids are polyunsaturated fatty acid (PUFA) [8]. Although many wild mushrooms have been used for medicinal purposes because of their useful components and biological activities, consumer have not been able to eat these mushrooms because of their relative scarcity. To commercialize wild mushrooms, many studies have focused on the development of artificial cultivation methods using various materials and conditions [9].

In this study, fatty acid composition of six wild edible mushroom species collected from different regions of Anatolia were analyzed to evaluate their nutritional values. 

## 2. Materials and Methods

### 2.1. Samples

In this study, six wild edible mushroom species (*Boletus reticulatus *Schaeff., *Flammulina velutipes *(Curtis) Singer var. *velutipes*, *Lactarius salmonicolor *R. Heim & Leclair, *Pleurotus ostreatus *(Jacq.) P. Kumm., *Polyporus squamosus *(Huds.) Fr., and *Russula anthracina *Romagn.) were analyzed for their fatty acid compositions. Origin and habitat information of these macrofungi has been given in Table 1. All of the analyzed mushrooms were identified as edible macrofungi belonging to class Basidiomycetes. The samples of the above six species were collected from different regions of Anatolia. All macrofungi samples were deposited in the Biology Department, Celal Bayar University, Turkey.

### 2.2. Fatty Acid Composition

The dried mushroom samples were powdered to ~1 mm particle size and used for analysis. Fatty acids were determined by gas-liquid chromatography with flame ionization detection (GLC-FID)/capillary column based on the ISO 5509 [10] *trans*-esterification method. The fatty acid profile was analyzed with a Chrompack CP 9001 chromatograph equipped with a split-splitless injector, a FID and a Chrompack CP-9050 autosampler. The temperatures of the injector and detector were 250°C. Separation was achieved on a 50 m × 0.25 mm i.d. fused silica capillary column coated with a 0.19 *μ*m film of CP-Sil 88. Helium was used as carrier gas at an internal pressure of 120 kPa. The column temperature was 140°C, for a 5 min hold, and then programmed to increase to 220°C at a rate of 4°C/min and then held for 10 min. The split ratio was 1 : 50, and the injected volume was 1.2 *μ*L. The results are expressed in relative percentage of each fatty acid, calculated by internal normalization of the chromatographic peak area. Fatty acid identification was made by comparing the relative retention times of FAME peaks from samples with standards. A Supelco mixture of 37 FAMEs (standard 47885-U) was used. Some fatty acid isomers were identified with individual standards also purchased from Supelco [1].

### 2.3. Statistical Analysis

The data presented are the averages of the results of three replicates with a standard error of less than 5%.

## 3. Results and Discussion

The fatty acid compositions of the wild edible mushrooms analyzed are shown in Table 2. 

In the present work, fatty acid compositions of fruit bodies of six wild edible mushroom species (*Boletus reticulatus*, *Flammulina velutipes *var. *velutipes*, *Lactarius salmonicolor*, *Pleurotus ostreatus*, *Polyporus squamosus,* and *Russula anthracina*) were investigated. The fatty acid compositions were different among all species. Unsaturated fatty acid levels were higher than saturated ones. This agrees with the observations that unsaturated fatty acids predominate over saturated ones in mushrooms [11]. The carbon chain lengths of fatty acids were from 4 to 24. *cis*-Linoleic acid was the major fatty acid detected in all species. In addition to *cis*-linoleic acid, *cis*-oleic, palmitic, and stearic acids were the other abundant fatty acids in the mushrooms. These four fatty acids were present in all of the mushrooms examined. Similar observations have been made in other mushrooms [1, 2].

All the mushrooms analyzed contained large quantities of essential fatty acid, *cis*-linoleic acid. Essential fatty acids are fatty acids that humans and other animals must ingest because the body requires them for good health but cannot syntesize them [12]. *cis*-Linoleic acid (18 : 2) was obtained in high amounts in *P. ostreatus* (65.29%). *cis*-Linoleic acid occurred in large amounts in the fruit bodies of *L. salmonicolor* (59.44%) and *F. velutipes* (40.87%) compared to other fatty acids. These results were in agreement with the previous reports that many mushroom species had high proportions of unsaturated fatty acids, especially linoleic acid [13, 14]. It is known that linoleic acid is the precursor of 1-octen-3-ol, known as the alcohol of fungi, which is the principal aromatic compound in most fungi and might contribute to mushroom flavour [15]. The percentages of *cis*-oleic acid in the fruit bodies of *R. anthracina*, *B. reticulatus, *and *P. squamosus* were 52.11, 37.61, and 33.02%, respectively. Fortunately, trans isomers of unsaturated fatty acids were detected in very low amounts (0.02–0.12%) in the studied mushrooms (Table 2). A rapidly expanding literature documents the importance of trans fatty acids (TFAs) in human health due to the increased risk of cardiovascular disease where they are negatively correlated with plasma HDL-cholesterol concentration and positively correlated with plasma LDL-cholesterol level [16]. It is also important to point out that, in contrast to other fungi [17, 18], no other fatty acids with an odd number of carbon atoms have been detected in considerable amounts. 

In previously studies, sixteen species of wild edible mushrooms found in Poland contained 66–82% linoleic acid and 10–20% palmitic acid, whereas lauric, myristic, stearic, arachidic, oleic, and palmitic acids were in smaller fractions [19]. Linoleic and palmitic acids were the predominant fatty acids of both glycolipids and phospholipids in *Pleurotus florida* [20]. Saturated and monounsaturated fatty acids were 20.2 and 63.9% in *P. ostreatus*, respectively [6]. Linoleic and palmitic acids were 63.7 and 18.6% in *F. velutipes*, respectively [2]. 

In general, approximately 70% of fatty acids were the same in all species included in this study. In addition, results indicated that mushrooms were rich in polyunsaturated fatty acids, especially 18 : 2. Also, mushrooms are rich in essential fatty acids and therefore should be contained in the diet.

## 4. Conclusion

Edible mushrooms can be regarded as healthy foods—low in fat. Low calorie and low fat diets are recommended for people with high blood cholesterol. Therefore mushrooms are perfect, because of their low calories, low fat composition, and high essential fatty acid levels [6]. Most of the studies on mushroom fatty acids are limited to certain mushroom species. However, the present results indicate that economically important wild edible mushrooms contain significant amounts of valuable fatty acids. Therefore, studies should be performed on fatty acid contents of other economically important and edible mushrooms.

## Figures and Tables

**Table 1:** Fungarium numbers and locality information of six wild edible mushroom species.

Species	Fungarium number	Coordinates	Localities
*Boletus reticulatus *	1091	N 41°03′-E 33°41′	Ilgaz Mountain
*Flammulina velutipes *	2127	N 39°56′-E 32°49′	Ankara
*Lactarius salmonicolor *	2413	N 40°36′-E 31°17′	Bolu
*Pleurotus ostreatus *	3093	N 39°56′-E 32°49′	Ankara
*Polyporus squamosus *	3091	N 39^0^56′-E 32°49′	Ankara
*Russula anthracina *	1184	N 41°06′-E 33°44′	Ilgaz Mountain

**Table 2:** Composition of fatty acids in six wild mushrooms (dry basis, % of total fatty acid).

	PS	PO	LS	FV	RA	BR
C4:0 butyric acid	0.03	0.06	0.09	0.05	0.01	0.06
C6:0 caproic acid	0.03	0.07	0.03	0.02	0.01	0.17
C8:0 caprilic acid	0.05	0.21	1.66	0.19	0.03	0.18
C10:0 capric acid	0.74	1.36	0.16	0.06	0.08	0.20
C11:0 undecanoic acid	0.09	0.07	0.03	0.04	0.10	0.87
C12:0 lauric acid	0.10	0.21	0.17	0.26	0.04	0.06
C13:0 tridecanoic acid	0.04	0.06	0.01	0.03	0.02	0.02
C14:0 myristic acid	0.58	0.66	0.16	0.50	0.15	0.37
C14:1 myristoleic acid	0.06	0.03	0.03	0.04	0.01	0.11
C15:0 pentadecanoic acid	0.89	1.99	0.35	0.76	0.35	0.37
C15:1 pentadesenoic acid	0.07	0.10	0.04	0.99	0.03	ND
C16:0 palmitic acid	17.21	12.40	7.76	14.56	9.16	11.26
C16:1 palmitoleic acid	0.65	0.46	0.22	0.70	0.64	0.75
C17:0 margaric acid	0.48	0.21	0.22	0.07	0.08	0.28
C17:1 heptadesenoic acid	0.16	0.04	0.03	0.06	0.08	0.99
C18:0 stearic acid	3.28	3.71	7.27	3.56	12.96	5.84
C18:1 *trans*-oleic acid	0.07	0.05	0.05	0.06	0.04	0.12
C18:1 *cis*-oleic acid	33.02	10.36	18.98	16.37	52.11	37.61
C18:2 *trans*-linoleic acid	0.09	0.06	0.03	0.05	0.03	0.02
C18:2 *cis*-linoleic acid	38.91	65.29	59.44	40.87	22.39	36.60
C18:3 linolenic acid	0.27	0.03	ND	0.01	0.01	ND
C18:3 gamma	0.26	0.26	1.40	17.25	0.03	0.31
C20:0 arachidic acid	0.30	0.13	0.45	0.19	0.21	0.58
C20:1 eicosenoic acid	0.11	0.11	0.03	0.08	0.18	0.50
C20:2 eicosadienoic acid	0.17	0.18	0.08	0.15	0.05	0.34
C21:0 heneicosanoic acid	0.06	0.06	0.06	0.04	0.05	0.30
C20:3 *n* = 3 *cis*-11,14,17-eicosatrienoic acid	ND	0.01	0.03	ND	ND	0.04
C20:4 arachidonic acid	0.04	0.02	ND	0.02	ND	0.11
C20:3 *n* = 6 *cis*-8,11,14-eicosatrienoic acid	0.10	0.02	0.05	0.06	0.02	ND
C22:0 behenic acid	0.43	0.11	0.14	0.04	0.04	0.08
C20:5 eicosapentaenoic acid	0.25	ND	0.24	0.47	0.24	0.45
C22:1 erucic acid	0.16	0.24	0.20	0.12	0.23	0.27
C22:2 docosadienoic acid	0.16	0.15	0.13	0.36	0.06	0.23
C23:0 tricosanoic acid	0.42	0.02	0.03	0.03	0.02	0.03
C24:0 lignoseric acid	0.52	0.48	0.42	0.30	0.43	0.51
C24:1 nervonic acid	ND	ND	ND	0.16	ND	ND
C22:6 docosahexaenoic acid	0.41	0.48	0.19	1.43	0.13	0.32

**SFA **(saturated fatty acids)	25.19	21.77	18.96	20.67	23.72	21.14
**MUFA **(monounsaturated fatty acids)	34.27	11.37	19.56	18.58	53.30	40.34
**PUFA** (polyunsaturated fatty acids)	40.64	66.48	61.57	60.65	22.94	38.40

PS: *Polyporus squamosus*; PO: *Pleurotus ostreatus*; LS: *Lactarius salmonicolor*; FV: *Flammulina velutipes*; RA: *Russula anthracina*; BR: *Boletus reticulatus*; ND: not determined.
